# Therapeutic efficacy against *Mycobacterium tuberculosis* using ID93 and liposomal adjuvant formulations

**DOI:** 10.3389/fmicb.2022.935444

**Published:** 2022-08-26

**Authors:** Susan L. Baldwin, Valerie A. Reese, Sasha E. Larsen, Tiffany Pecor, Bryan P. Brown, Brian Granger, Brendan K. Podell, Christopher B. Fox, Steven G. Reed, Rhea N. Coler

**Affiliations:** ^1^Seattle Children’s Research Institute, Center for Global Infectious Disease Research, Seattle, WA, United States; ^2^Access to Advanced Health Institute, Seattle, WA, United States; ^3^Department of Microbiology, Immunology and Pathology, Colorado State University, Fort Collins, CO, United States; ^4^Department of Global Health, University of Washington, Seattle, WA, United States; ^5^HDT Bio Corp., Seattle, WA, United States; ^6^Department of Pediatrics, University of Washington School of Medicine, Seattle, WA, United States

**Keywords:** adjuvant, tuberculosis, immunotherapy, QS-21, glucopyranosyl lipid adjuvant

## Abstract

*Mycobacterium tuberculosis* (*M.tb*) has led to approximately 1.3 million deaths globally in 2020 according to the World Health Organization (WHO). More effective treatments are therefore required to prevent the transmission of *M.tb*. Although Bacille Calmette–Guérin (BCG), a prophylactic vaccine against *M.tb*, already exists, other vaccines are being developed that could help boost BCG’s noted incomplete protection. This includes ID93 + GLA-SE, an adjuvanted protein vaccine which is being tested in Phase 2 clinical trials. The aim of this study was to test new lipid-based adjuvant formulations with ID93 in the context of a therapeutic vaccine, which we hypothesize would act as an adjunct to drug treatment and provide better outcomes, such as survival, than drug treatment alone. The recent success of another adjuvanted recombinant protein vaccine, M72 + AS01_E_ (GlaxoSmithKline Biologicals), which after 3 years provided approximately 50% efficacy against TB pulmonary disease, is paving the way for new and potentially more effective vaccines. We show that based on selected criteria, including survival, T helper 1 cytokine responses, and resident memory T cells in the lung, that a liposomal formulation of GLA with QS-21 (GLA-LSQ) combined with ID93 provided enhanced protection over drug treatment alone.

## Introduction

The annual number of deaths from tuberculosis (TB), caused by *Mycobacterium tuberculosis* (*M.tb*), has been surpassed by COVID-19, caused by the Severe Acute Respiratory Syndrome Coronavirus-2 (SARS-CoV-2) which, beginning in December of 2019, resulted in a pandemic. As of April 2022, the SARS-CoV-2 virus has claimed the lives of more than 6 million people (Johns Hopkins Corona Resource Center website; coronavirus.jhu.edu/). This has severely impacted the treatment of TB patients, through the redirection of resources and services to combat COVID-19 (Togun et al., 2020; WHO, Global Tuberculosis Report, 2020, 2021). The negative impact on TB care includes budget reallocation from TB to COVID-19, delays in patients seeking care due to fear of COVID-19, delays in treatment due to overburdened healthcare providers and facilities, and delays in diagnosis and treatment courses due to the repurposed use of GeneXpert machines in high TB-burden countries (Togun et al., 2020; WHO, Global Tuberculosis Report, 2020). The decrease in TB case notifications is staggering, from 7.1 million in 2019 to 5.8 million in 2020 (WHO, Global Tuberculosis Report, 2021). The number of people estimated to have died from TB has risen to 1.3 million in 2020, from 1.2 million reported in 2019, and the consequences of the COVID-19 pandemic are expected to have an even more dire impact on the number of estimated deaths due to TB in 2021 (WHO, Global Tuberculosis Report, 2021). Now more than ever, new therapeutic strategies to improve the course of TB treatment are needed. Six months of first-line drug treatment has typically been given for therapy against drug-susceptible *M.tb*; this regimen includes the use of four drugs: Rifampicin (RIF), Isoniazid (INH), Pyrazinamide (PZA), and Ethambutol (EMB) for the first 2 months, followed by RIF and INH for the next 4 months (Nahid et al., 2016). The global treatment success rate of new TB cases was 86% in 2019 according to the 2021 WHO global tuberculosis report (WHO, Global Tuberculosis Report, 2021). A 4-month Rifapentine-moxifloxacin TB treatment regimen is now also an option for the treatment of drug-susceptible pulmonary TB,^1^ where the shortened regimen was shown to be non-inferior to the standard 6-month regimen (Dorman et al., 2021). More troubling are cases involving drug resistance (DR) such as Rifampin-resistant (RR) and multiple drug resistant (MDR) strains of *M.tb*, which require a second-line drug regimen for a minimum of 9 and up to 20 months. Furthermore, the successful global treatment rate of MDR-TB and RR-TB in 2018, while improving over time since 2012, is still extremely low at 59% (WHO, Global Tuberculosis Report, 2021). We have demonstrated in a preclinical model that the use of ID93 + glucopyranosyl lipid adjuvant formulated in an oil-in-water emulsion (GLA-SE) was successfully given as an adjunct to drug treatment to further reduce bacterial burden and maintain functional pulmonary architecture with a reduced antibiotic regimen (Coler et al., 2013; Larsen et al., 2018). GLA is a synthetic Toll-like receptor (TLR4) agonist, which has been described elsewhere (Coler et al., 2011). Here, we expand on our therapeutic TB vaccine work by investigating the use of vaccine candidate ID93 with liposomal formulations of GLA to determine its therapeutic efficacy potential. Additional effective adjuvant formulations will be required to fulfill the growing need of potent vaccines, particularly against diseases such as TB. An effective, readily available therapeutic vaccine given in conjunction with drug treatment could work to prevent active disease and transmission of this pathogen which could dramatically reduce the morbidity and mortality associated with *M.tb* infections worldwide.

## Materials and methods

### Antigen and adjuvants

The ID93 recombinant fusion protein is comprised of the *M.tb* proteins Rv2608, Rv3620, Rv1813, and Rv3619 and was prepared at the Infectious Disease Research Institute (IDRI), now called Access to Advanced Health Institute (AAHI; Seattle, WA), as previously described (Bertholet et al., 2010). Liposomal formulations of GLA were prepared as previously published (Baldwin et al., 2021). GLA was obtained from Avanti Polar Lipids and was formulated in an aqueous nanosuspension (GLA-AF), as previously described (Misquith et al., 2014), or in two different liposome compositions. The liposome compositions were prepared as follows: anionic liposomes {GLA-LS2 [(3.3:0.4:1 weight ratio of DPPC:DPPG:cholesterol in 25 mM ammonium phosphate buffer)]} or neutral liposomes [GLA-LS3 (4:1 weight ratio of DOPC:cholesterol in 25 mM ammonium phosphate buffer)]. DPPC (1,2-dipalmitoyl-sn-glycero-3-phosphocholine) and DPPG [1,2-dipalmitoyl-sn-glycero-3-phospho(1′-rac-glycerol)] were obtained from Lipoid LLC, Newark, NJ, United States. DSPE-PEG2000 (1,2-distearoyl-sn-glycero-3-phosphoethanolamine-N-[methoxy(polyethylene glycol)-2000)] was purchased from CordenPharma International. Plant-derived cholesterol was acquired from Sigma-Aldrich Fine Chemicals (SAFC), St. Louis, MO, United States and buffer salts were obtained from J.T. Baker (Phillipsburg, NJ). In addition, each liposome composition was optionally formulated with QS-21, prepared at IDRI (Seattle, WA) from semi-purified saponin (Quil A from Brenntag Biosector, Frederikssund, Denmark), or obtained directly from Desert King International (San Diego, CA), by adding aqueous QS-21 to the prepared liposome (e.g., GLA-LSQ2 for anionic QS-21 liposomes, GLA-LSQ3 for neutral QS-21 liposomes). QS-21 is an extract derived from the *Quillaja saponaria* soap bark tree (Del Giudice et al., 2018; Lacaille-Dubois, 2019). In general, liposomes were prepared as 2 or 4x concentrate and mixed with antigen and diluent prior to administration.

### Animals

Female SWR mice, 5–7 weeks old, were purchased from Jackson Laboratories (Bar Harbor, Maine). Mice were maintained under specific pathogen-free conditions in the IDRI animal facility, and housed in BL3 containment after infection. Mice were treated in accordance with the regulations and guidelines of the IDRI Institutional Animal Care and Use Committee (IACUC).

The IDRI IACUC approved the protocol for these animal studies. Mice used in these experiments were treated in accordance with the regulations and guidelines of the IACUC (Protocol Numbers: 2011–5, 2014–9, and 2017–9) and with recommendations from the National Institute of Health Guide for the Care and Use of Laboratory Animals. The method of euthanasia used is consistent with the recommendation of the Panel on Euthanasia of the American Veterinary Medical Association. Mice were ethically sacrificed by controlled administration of inhalation of carbon dioxide followed by cervical dislocation.

### Infection

Mice were infected with a low dose aerosol (LDA) of *M. tuberculosis* (*M.tb*) H37Rv using an aerosol exposure chamber (University of Wisconsin, Madison, WI, United States) calibrated to deliver 50–100 CFU into the lungs of each mouse. Twenty-four hours after infection of mice, three animals were euthanized and entire lung homogenates were plated on Middlebrook 7H10 agar (Molecular Toxicology, Inc., Boone, NC, United States) to enumerate bacteria delivered. An average of 46 CFU was delivered per mouse.

### Antibiotic treatment

Four weeks after infection, an antibiotic regimen of Rifampicin (R; Chem Impex International, Wood Dale, IL), Isoniazid (H), and Pyrazinamide (Z; both Acros Organics, subsidiary of Thermo Fisher, Waltham, MA, United States) was initiated in the drinking water for 8 weeks. Doses were 100 mg/L (R), 250 mg/L (H), and 150 mg/L (Z).

### Immunizations

Starting 8 weeks after aerosol infection (4 weeks after initiation of antibiotic regimen) mice were immunized intramuscularly with saline (unimmunized control group) or 0.5 ID93 admixed with specified adjuvants, three times at 3-week intervals.

### Bacterial burden

A subset of mice were euthanized 4 weeks after final therapeutic immunization. The lung (except for the accessory lobe, which was used for histology) and spleen were homogenized in RPMI using an Omni Tissue Homogenizer and soft tissue probes (Omni International, Kennesaw, GA, United States). Serial dilutions of organ homogenates were made in PBS with 0.05% Tween80 (Sigma, St. Louis, MO, United States), and aliquots of dilutions were plated on Middlebrook 7H10 agar plates, with remaining homogenates used for flow cytometry. Plates were incubated for 3 weeks at 37°C, 5% CO_2_, before colony enumeration. Bacterial burden, in colony forming units (CFU) per organ, was calculated and expressed as Log_10_. Reduction in bacterial burden was calculated as (Mean Log_10_ CFU_saline_ – Mean Log_10_ CFU_vaccine_).

### Flow cytometry

Cells from mouse lung or spleen homogenates were resuspended in RPMI1640 (Life Technologies, Carlsbad, CA) with 10% FBS (Sigma) with pen/strep (Life Technologies) and glutamine (Gemini), and dispensed into 96-well round bottom plates. For intracellular cytokine production, cells were stimulated with medium alone, ID93 at 10 μg/ml, or *M.tb* lysate (BEI) at 1 μg/ml for 2 h at 37°C as described in the figure legends. Subsequently, Brefeldin A at 1 μg/ml (GolgiPlug; BD Biosciences, San Jose, CA, United States) was added, and samples were incubated for an additional 8 h at 37°C. Plates were held at 4°C overnight before staining with antibodies.

Stimulated cells were incubated with fluorochrome-conjugated monoclonal antibodies to mouse CD4 (clone RM4-5, eBioscience, San Diego, CA), CD8 (clone 53–67, Biolegend, San Diego, CA), and CD44 (clone IM7, eBioscience) in 1% BSA in PBS with 1 μg/ml Fc Block (CD16/CD32, Clone 93, 138 eBioscience) for 10 min at room temperature. After washing, cells were treated with Cytofix/Cytoperm (BD Biosciences) for 20 min at room temperature, and then washed with Perm/Wash buffer (BD Biosciences). Intracellular staining was done with fluorochrome-conjugated monoclonal antibodies to GM-CSF (clone MP1-22E9), IL-5 (clone TRFK5), and IL-17 (clone TC11-18H10.1), all from BioLegend, plus CD154 (clone mr1), IL-2 (clone JESS-5H4), IFNγ, TNFα (clone MP6-XT22), and IL-21 (clone mhalx21) from eBioscience in Perm/Wash buffer. All antibodies were used at 1:100 dilution. Stained cells were washed and resuspended in 1% BSA in PBS, and filtered before analysis on a modified 4 laser Fortessa with FACSDiva software (BD Biosciences). Lymphocytes were gated by forward and side scatter and the gating strategy for intracellular cytokines are shown from the same animal following stimulation with either ID93 (Supplementary Figure 1A) or medium (Supplementary Figure 1B). Gating strategies are also shown for effector memory T cells (Tem), central memory T cells (Tcm), and resident memory T cells (Trm; Supplementary Figure 2A), and for CD25 + FoxP3 + CD4 T cells (Supplementary Figure 2B). Data were analyzed with FlowJo version 9.9.6 (Treestar, Ashland, OR) and SPICE (National Institutes of Health, http://exon.niaid.nih.gov/spice).

### Antibody ELISA

Mice were bled for serum at time points as described in the figure legends and the ELISAs were performed as previously described (Baldwin et al., 2021). Briefly, plates were coated overnight at 4°C with 2 μg/ml of ID93, Rv2608, Rv3620, Rv1813, or Rv3619 in 0.1M bicarbonate, followed by blocking with PBS/0.05% Tween20/1% Powdered Skim Milk and washing the plates. Sera were diluted in PBS/0.05% Tween20/0.1%. Powdered Skim Milk using serial 5-fold dilutions and were added to the plates for 2 h at RT. Plates were washed, and HRP-conjugated goat anti-mouse IgG1, IgG2c, or total IgG antibodies (Southern Biotech, Birmingham, AL) at 1:2,000 dilution for IgG1 and IgG2c or 1:4,000 for total IgG were added to the plates, with incubation of 1 h at RT. Plates were washed and SureBlue tetramethylbenzidine substrate solution (Seracare) was added. The reaction was stopped with 1 N H_2_SO_4_ (Fisher) and plates were read on a Synergy2 microplate reader (BioTek, Winooski, VT) at 450 nm with 570 nm background subtraction. Reciprocal dilutions corresponding to endpoint titers were determined with GraphPad Prism 8.1 (GraphPad Software, San Diego, CA) with a cutoff value of naïve serum control wells +2SD. Samples with absorbance too low to calculate an endpoint were assigned a value of zero.

### Survival

Survival was monitored in 9–11 mice per group following *M.tb* infection. Animals with greater than 20% weight loss, or moribund condition, were euthanized. Bacterial burden in lungs and spleens was determined as described above.

### Histology

A subset of mice were euthanized 4 weeks after final therapeutic immunization for histology assessment. Four weeks after the last therapeutic immunization, the accessory lung lobes, in their entirety, from 4 mice per group, were stored in neutral buffered formalin (NBF) for fixation, placed in a tissue cassette, embedded in paraffin by the University of Washington histology core, and sent to Colorado State University for average lesion percentage scores. The tissues were sectioned (one section per mouse) and stained by a histotechnician external to this study to ensure that there was no introduced bias in the analysis. Any variability was therefore considered attributable as a result of disease extent and distribution in each of the accessory lobes. For the staining procedure, 5 μm sections were mounted and stained with H&E following standard procedure. H and E-stained slides were scanned at X20 using Olympus VS120 microscope. Image analysis was performed using VisioPharm image analysis software (Horsholm, Denmark). For each tissue section, a region of interest (ROI) was generated at a low magnification with a custom tissue detecting algorithm using decision forest training and classification to differentiate tissue versus background based on color and area. Lesions were identified within tissue ROI’s at a high magnification with an additional custom-made algorithm using decision forest training and classification based on staining intensity, color normalization and deconvolution, area, and morphological features. Percent of lung area occupied by lesion pathology was calculated by the ratio of ROI areas to total area of each lung lobe. Lesion identification and quantification were then reviewed by a pathologist and edited, if necessary.

### Statistical analysis

Bacteria burden, single-cytokine-producing T cells, polyfunctional-cytokine-producing T cells, antibody ELISA endpoint titers, memory population, and PD1/KLRG1 subsets were assessed versus control groups specified in figures using one-way ANOVA with Bonferroni’s multiple comparisons correction. The above analyses were performed with GraphPad Prism 8.1 (GraphPad Software, San Diego CA, United States).

Survival analysis was performed in R version 3.14 using the Survival package (Therneau, 2015) The effect, significance, and hazard for each intervention were determined relative to the untreated control group using a Cox Proportional Hazard model. Kaplan–Meier survival and cumulative hazard curves were visualized using the ggplot package (Wickham, 2016).

## Results

### Use of GLA-liposomal formulations as adjuvants for therapeutic use against *Mycobacterium tuberculosis*

**Figure 1:**
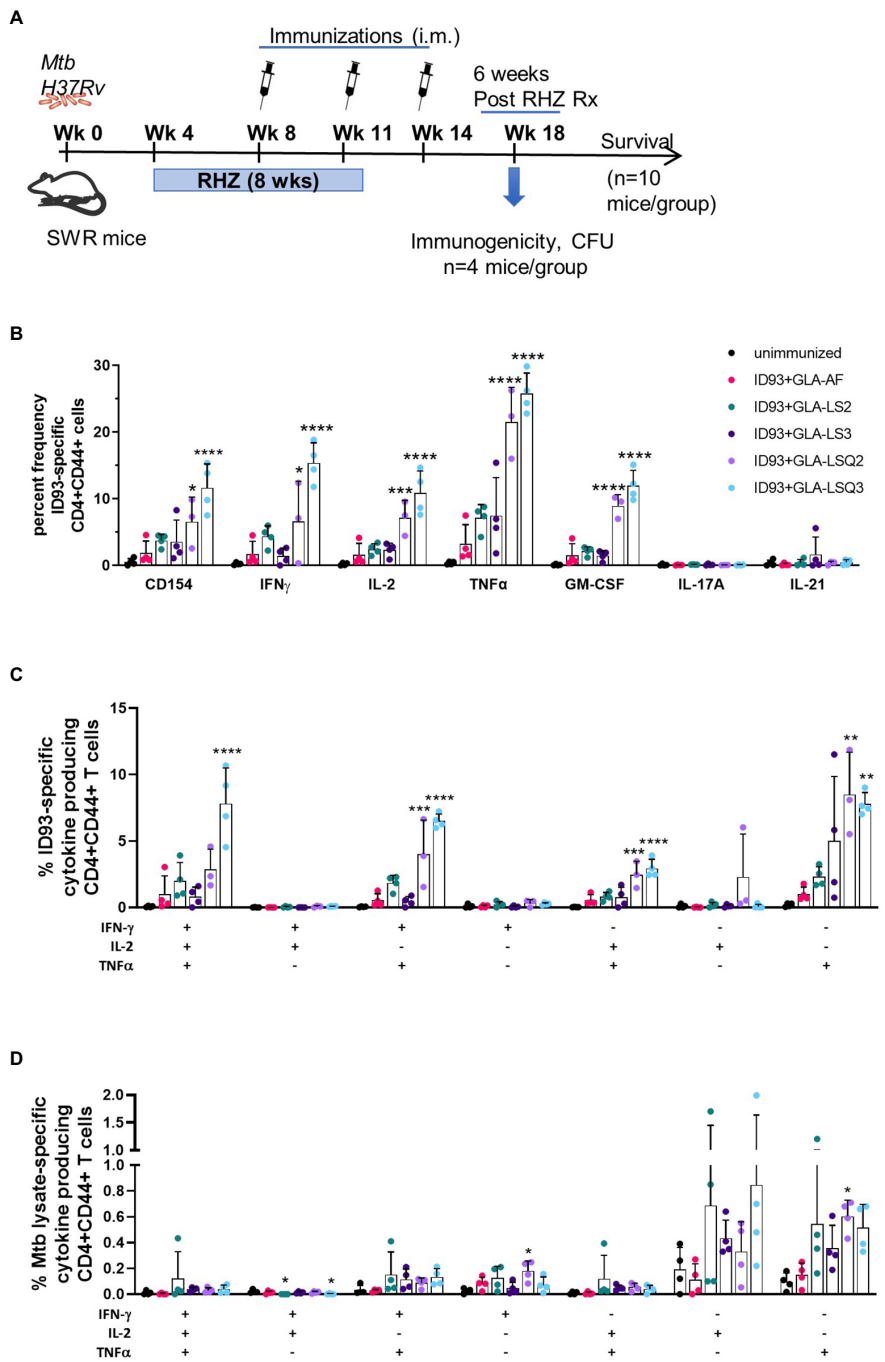
Enhanced immunogenicity in female SWR/J mice after treatment with drug and therapeutic ID93 vaccines. **(A)** Therapeutic study timeline; **(B)** Percent frequency of CD4 + CD44+ ID93-specific single-cytokine-producing cells; asterisks indicate significance where ^***^p < 0.001, ^****^p < 0.0001 using one-way ANOVA with Bonferroni’s multiple comparison correction; **(C)** ID93-specific CD4 + CD44+ polyfunctional Th1 cytokine-producing cells; **(D)** *Mycobacterium tuberculosis* lysate-specific CD4 + CD44+ polyfunctional Th1 cytokine-producing cells. Dots represent individual mice, with bars as the mean, and whiskers indicating SD. Asterisks indicate statistical significance, where ^**^p < 0.01, ^***^p < 0.001, and ^****^p < 0.0001 using one-way ANOVA with Bonferroni’s multiple comparison correction.

Our main goal was to determine the therapeutic potential of ID93 plus GLA-containing liposomal adjuvant formulations, previously used in combination with ID93 as a prophylactic vaccine against *M.tb* (Baldwin et al., 2021). We have previously included other adjuvanted ID93-based vaccines in the SWR mouse model as an adjuvant to drug treatment against *M.tb* (Coler et al., 2013; Larsen et al., 2018). As shown in Figure 1A, SWR mice were challenged with *M.tb* H37Rv, then started on antibiotic treatment with Rifamycin (R), Isoniazid (H), and Pyrazinamide (Z), RHZ, 4 weeks later. Mice were treated with the antibiotic regimen in the drinking water for 8 weeks. Four weeks after the initiation of drug treatment, mice were immunized three times, 3 weeks apart with ID93 combined with one of five different adjuvant formulations as recently described (Baldwin et al., 2021): briefly, these include GLA-AF (an aqueous formulation), GLA-LS2 (anionic liposome), GLA-LS3 (neutral liposome), GLA-LSQ2 (anionic liposome + QS-21), and GLA-LSQ3 (neutral liposome + QS-21).

### The ID93 + GLA-LSQ3 therapeutic vaccine candidate elicits a Th1 immune response

To determine ID93-specific immune responses within the lung, a subset of mice from each vaccine group were euthanized 4 weeks after the therapeutic vaccine boost, and 6 weeks after the end of drug treatment (EOT) utilizing flow cytometry and the gating strategy shown in Supplementary Figures 1A,B. As shown in Figure 1B, the activation marker CD154 was significantly elevated in mice immunized with ID93 + GLA-LSQ3 compared to the unimmunized (drug treatment only) group. When assessing Th1 cellular immunity, GLA-LSQ3 combined with ID93 elicited a significantly higher frequency of CD4 + CD44+ T cells expressing a Th1 cytokine response (including IFNγ, TNFα, and IL-2) compared to unimmunized mice (Figure 1B). Whereas the GLA-LSQ2 vaccine candidate induced CD44 + CD4 + TNFα and IL-2 responses, this vaccine did not induce significant levels of IFNγ (Figure 1B). Both of the ID93 + GLA-LSQ vaccines were able to induce GM-CSF (Figure 1B). Compared to the unimmunized (drug treatment only) group, there was a statistically significant increase in polyfunctional CD4 + CD44+ T cells with both ID93 + GLA-LSQ2 and ID93 + GLA-LSQ3, although only mice given ID93 + GLA-LSQ3 had triple Th1 cytokine-expressing (IFNγ, TNFα, and IL-2) CD4+ T cells (Figure 1C). Polyfunctional ID93-specific CD4+ T cells expressing two or more cytokines (which included the CD154 activation marker and/or four cytokines including IFNγ, TNFα, IL-2, and 227 GM-CSF) were significantly increased in both of the ID93 GLA-LSQ vaccines compared to the other vaccine candidates; ID93+GLA-LSQ3 was significant compared to ID93+GLA-LSQ2 (Supplementary Figure 3A); however, there were no significant differences in *M.tb* lysate-specific polyfunctional CD4+ T cells (Supplementary Figure 3B). Similarly, no statistical differences in polyfunctional CD4+ T cell responses following stimulation with *M.tb* lysate were observed in any of the groups in Figure 1D, although there was a slight trend to higher responses in mice immunized with ID93 + GLA adjuvant formulated in liposomes, mostly due to TNFα responses. Only the ID93+GLA-LSQ2 group induced *M.tb* lysate-specific IFNγ and TNFα responses (Figure 1D).

### All ID93 **vaccine candidates containing** GLA **elicit an ID93-specific IgG2c response**

**Figure 2:**
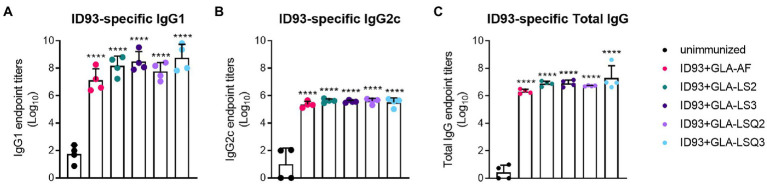
ID93-specific humoral immunity in SWR mice. ID93-specific IgG subclass responses. **(A)** ID93-specific IgG1; **(B)** ID93-specific IgG2c; and **(C)** ID93-specific total IgG responses in SWR mice following therapeutic vaccination with different formulations of GLA. All of the adjuvanted therapeutic vaccinations induced significant levels of each subclass of IgG. Dots represent individual mice, with bars as the mean, and whiskers indicating SD. Asterisks indicate significance (*p* < 0.0001) using one-way ANOVA with Bonferroni’s multiple comparisons correction.

As humoral responses could play a role in protection against *M.tb*, we next measured antigen-specific IgG1 and IgG2c, and total IgG responses to ID93 and to each of the four components of ID93: Rv2608, Rv1813, Rv3619, and Rv3620. All of the ID93 + “GLA-containing” adjuvants induced significant levels of IgG1 or IgG2c to ID93 (Figure 2) and each of the components of ID93 (Supplementary Figures 4A–D) compared to the unimmunized group (*p* < 0.05) regardless of the adjuvant formulation.

### Enhanced resident memory CD4 T cells, and multiple stages of differentiated CD4+ T cells occur in the lungs of mice immunized with ID93 vaccine candidates combined with GLA-LSQ adjuvants

**Figure 3:**
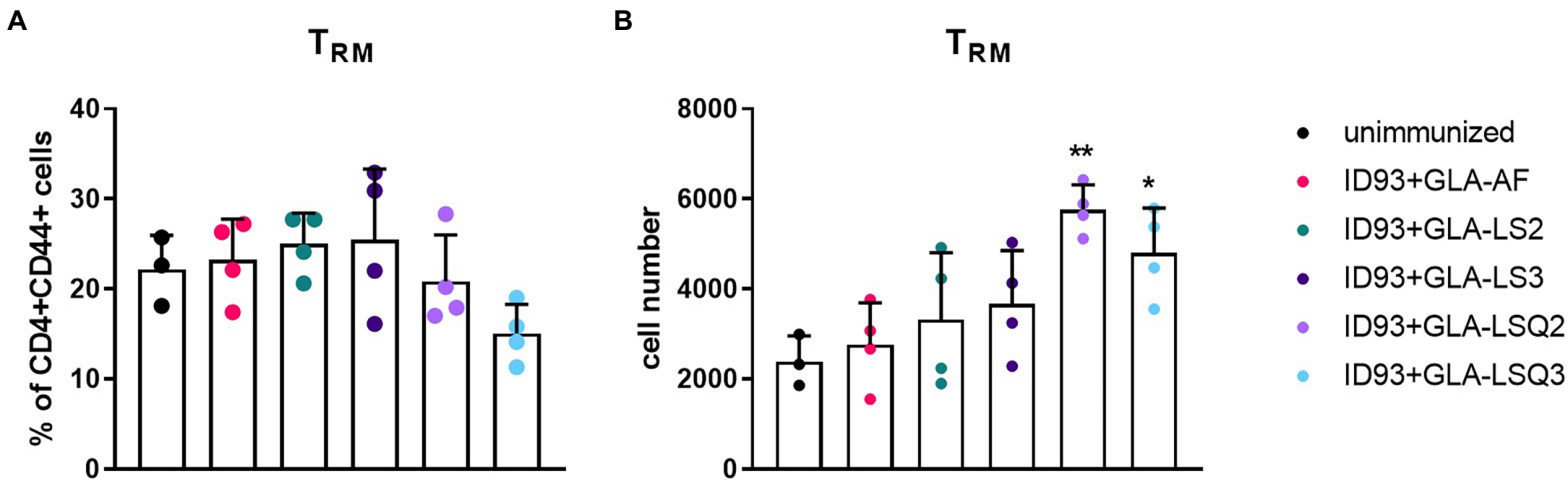
Enhanced numbers of resident memory T cells were observed in the lungs of mice immunized with ID93 combined with GLA liposomes and QS-21. Mice were immunized with the ID93 therapeutic vaccines 4 weeks after the start of drug treatment, three times, 3 weeks apart. Four weeks after the last therapeutic vaccination, mice (*n* = 4 mice/group) were euthanized, and lungs were harvested for flow cytometric analysis. **(A)** Percentage of CD4 + CD44+ resident memory T cells (Trm); **(B)** Absolute number of CD4 + CD44+ resident memory T cells (Trm). Dots represent individual mice, with bars as the mean, and whiskers indicating SD. Asterisks indicate statistical significance (^*^p < 0.05, ^**^p < 0.01) in mice given therapeutic immunization versus no immunization, using one-way ANOVA with Bonferroni’s multiple comparisons test.

We next wished to determine whether there were differences in the numbers of resident memory T cells (Trm) within the lungs of mice given immunotherapy with the ID93 vaccines in combination with drug treatment. Four weeks after the third immunotherapeutic vaccination, four mice per group were euthanized and lungs were harvested for flow cytometric analysis using the gating strategy in Supplementary Figure 2A. Although we did not observe increases in the percentage of Trm CD4+ T cells (CCR7-CD62L-CD69+) in the lungs in any of our therapeutic vaccine groups compared to the unimmunized group (Figure 3A), we did see a significant increase in the numbers of Trm CD4+ T cells in the lungs of mice given therapeutic vaccinations with ID93 combined with the GLA-LSQ adjuvants (GLA-LSQ2 and GLA-LSQ3; Figure 3B). We also observed no differences in the percentage or number of regulatory (CD4 + CD25 + FoxP3+) T cells (for gating strategy see Supplementary Figure 2B) in any of the groups except the ID93 + GLA-LS2 immunized group, where an increase in the percentage of CD4 + CD25 + FoxP3+ T cells was observed, but not the absolute number of regulatory T cells, (Supplementary Figure 5).

**Figure 4:**
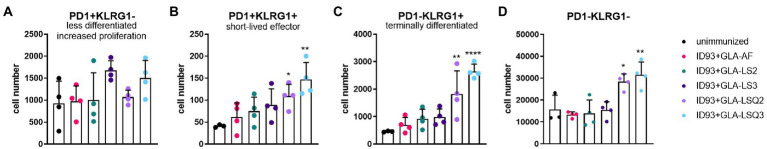
Multiple stages of differentiated CD4+ T cells in the lungs in mice immunized with ID93 combined with GLA liposomes (either neutral or anionic) combined with QS-21. Four weeks after the last therapeutic vaccination, mice (*n* = 4 mice/group) were euthanized, and lungs were harvested for flow cytometric analysis. Dots represent individual mice, with bars as the mean, and whiskers indicating SD. Asterisks indicate statistical significance (^*^p < 0.05, ^**^p < 0.01, ^****^p < 0.0001) in mice given therapeutic immunization versus no immunization, using one-way ANOVA with Bonferroni’s multiple comparisons correction. **(A)** PD1 + KLRG1—(less differentiated, increased proliferation); **(B)** PD1 + KLRG1+ (short-lived, cytokine-producing, effector T cells); **(C)** PD1-KLRG1+ (terminally differentiated, cytokine-producing T cells); and **(D)** PD1-KLRG1−.

To determine the state of differentiated CD4+ T cells in the lungs of each of the treatment groups, we included programmed cell death protein-1 (PD1) and killer cell lectin-like receptor subfamily-G1 (KLRG1) markers (gating strategy as shown in Supplementary Figure 2A). We included CD4 + CD44+ T cells that expressed these differentiation markers without utilization of a tetramer stain, therefore the responses are not antigen-specific. Even so, the lung cells from the ID93 + GLA-LSQ2 and ID93 + GLA-LSQ3 vaccine groups showed evidence of multiple stages of CD4+ T cell differentiation depending on PD1 and KLRG1 expression (Figure 4; Supplementary Figure 6), as previously defined by Reiley et al. (2010). For both immunotherapeutic vaccines, significant numbers of CD4+/CD44+ T cell subsets were observed including PD1 + KLRG1+ (short-lived, cytokine-producing, effector T cells) and PD1-KLRG1+ (non-proliferating, cytokine-producing, terminally differentiated T cells; Figure 4). Absolute numbers of the less differentiated CD4+ T cells, depicted by PD1 + KLRG1-, were not increased compared to unimmunized mice, in any of the vaccine-treated groups; however, the ID93 + GLA-LSQ vaccine groups were slightly trending higher in numbers compared to the unimmunized group (Figure 4A). This CD4 T cell data, with increased surface expression of KLRG1 (with cytokine-producing capability), follow nicely with our cytokine data (Figure 1), in which the vaccines containing the GLA-LSQ formulations induced the highest magnitude of antigen-specific cytokine-producing CD4+ T cells.

### Enhanced survival in animals therapeutically immunized with ID93+GLA-LSQ3 and ID93+GLA-LS2

**Figure 5:**
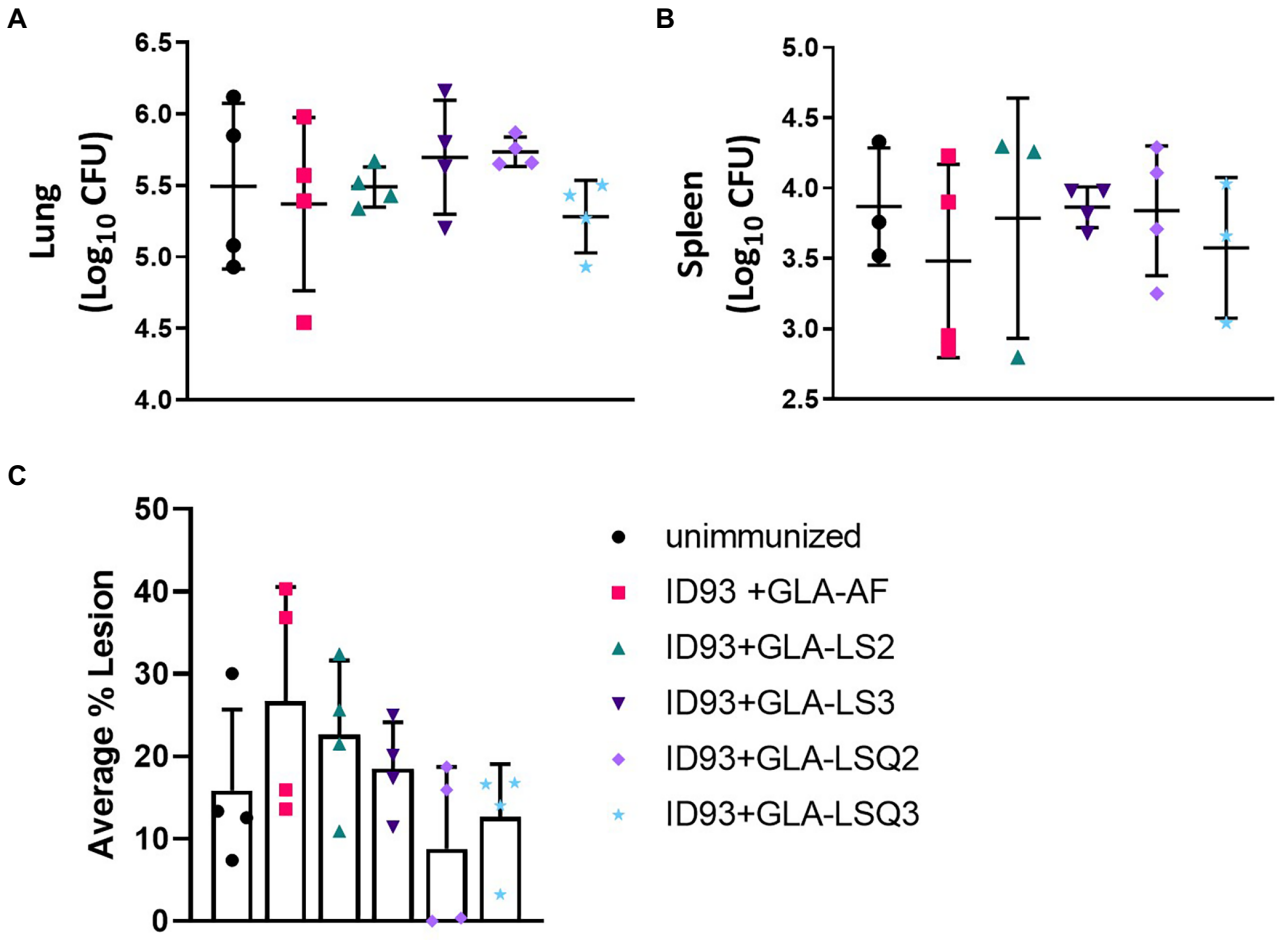
Bacterial burden in the lung/spleen and average percentage of lesions in the lungs of *M.tb* H37Rv-infected SWR mice 4 weeks following RHZ treatment and ID93 therapeutic vaccines. Four weeks after the last therapeutic vaccination, lungs and/or spleens mice (*n* = 4 mice/group) were harvested for bacterial burden assessment (lungs and spleen) or were collected for histology (lungs). Bacterial burden is represented as Log_10_ colony forming units (CFU) 4 weeks after the last therapeutic immunization (week 18 CFU) in the **(A)** lung or **(B)** spleen (*n* = 3 animals rather than 4 were included for ID93 + GLA-LS2 and ID93 + GLA-LSQ3 due to contamination of one of the mouse samples; only three animals were included in the unimmunized group as one animal was excluded as an outlier); in **(A,B)**, crossbars indicate the mean, whiskers indicate SD, and dots indicate individual mice; **(C)** the average percentage of lung lesions are shown. Dots represent individual mice, the bar is the mean, and whiskers indicate SD. No statistical significance was observed in any of the experimental groups compared to the unimmunized group by two-tailed unpaired *t*-test.

Four weeks following the last therapeutic vaccination, subsets of four animals per group were assessed for bacterial burden within the lungs and spleens in addition to the percentage of lesions within the lung. While there was no statistical significance between any of the groups either in reduction of bacteria in either organ (Figures 5A,B), or reduced percentage of lesions within the lungs (Figure 5C), this did confirm that the treatments were safe and did not exacerbate the infection or immunopathology at this timepoint. Both of the ID93 + GLA-LSQ therapeutic vaccines were observed to have less than 20% lesion averages within the lungs in all four mice whereas other vaccines showed more variable lesion averages.

**Figure 6:**
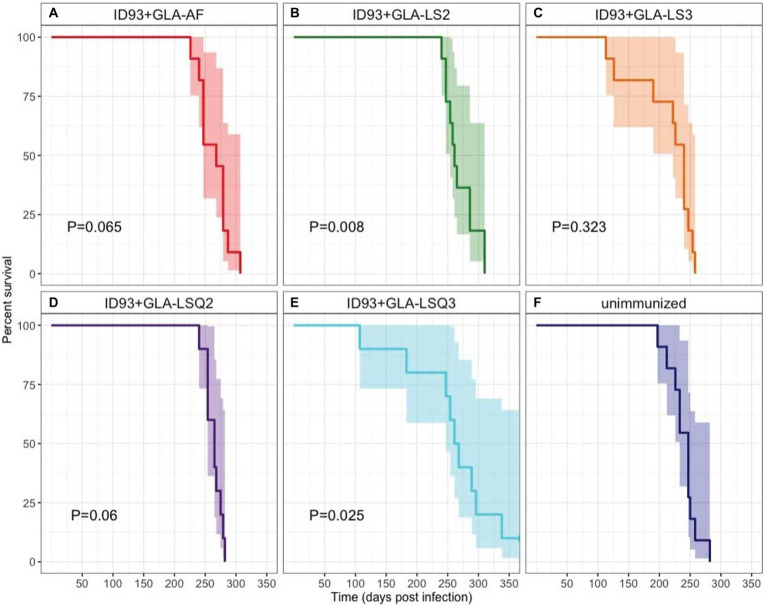
Kaplan–Meier survival curves for each intervention. A subset of 11 mice per group were followed for survival following *Mtb* H37Rv infection, treatment for 8 weeks with Isoniazid, Rifampin, and Pyrazinamide in the drinking water, and therapeutic immunization with ID93 vaccines 4 weeks after the start of drug treatment. 95% CI is indicated in the shaded region on each side of the Kaplan–Meier survival curve. Indicated *p* values were determined using Cox Proportional Hazard models and are relative to the unimmunized group. *N* = 11 mice per group, except for ID93 + GLA-LSQ2 and ID93 + GLA-LSQ3 (*n* = 9 mice/group); **(A)** Drug treatment + ID93 + GLA-AF; **(B)** Drug treatment + ID93 + GLA-LS2; **(C)** Drug treatment + ID93 + GLA-LS3; **(D)** Drug treatment + ID93 + GLA-LSQ2; **(E)** Drug treatment + ID93 + GLA-LSQ3; and **(F)** Drug treatment only (unimmunized).

Survival of 9–11 SWR mice per group was assessed over 350 days as a primary readout to determine whether immunotherapy with different ID93 vaccine formulations, given as an adjunct to 8 weeks of RHZ drug treatment, was effective at reducing morbidity and mortality from TB disease (Figure 6). The only two immunotherapeutic vaccines that significantly increased survival over drug treatment were vaccines with the anionic liposomal formulation, ID93 + GLA-LS2 (*p* = 0.008; Figure 6B) and with the QS-21-containing neutral liposomal formulation, ID93 + GLA-LSQ3 (*p* = 0.025; Figure 6E). Similarly, these interventions were also associated with the lowest hazard (ID93 + GLA-LS2: HR = 0.2708; ID93 + GLA-LSQ3: HR = 0.3035), relative to unimmunized (Supplementary Figure 7).

At the time of euthanasia (which occurred at different time frames) in the survival cohort, individual CFUs were determined in both the lung and spleen, and were assessed per intervention group at the end of the study. In the spleen, a significant decrease in bacterial load was observed in mice treated with ID93 + GLA-LSQ2 versus the ID93 vaccine with GLA-LS2 (without QS-21; Supplementary Figure 8). Decreased bacterial load in the lung was observed with ID93 + GLA-AF (aqueous formulation) and ID93 + GLA-LSQ2 (Supplementary Figure 8) compared to drug treatment alone. The lungs and spleens that were assessed for bacterial enumeration in Supplementary Figure 8 were collected at various timepoints at euthanasia. Although there was a cumulative decrease in bacterial load with ID93 + GLA-LSQ2 in the lung at euthanasia compared to the control (“drug only” group), this group did not show a significant increase in survival compared to the “drug only” group.

## Discussion

One of our main goals is to develop a TB vaccine candidate that can be used as an adjunct to drug treatment to either enhance therapeutic outcomes or shorten the length of treatment against TB. We have previously shown the merit of using ID93 + GLA-SE as a therapeutic vaccine in mice and NHPs, where ID93 + GLA-SE provided enhanced protection or immunogenicity in the context of first-line drug treatment (Coler et al., 2013; Larsen et al., 2018). In the current study, we compared different GLA-liposomal adjuvants, including anionic or neutral liposomes without QS-21 (LS2 or LS3, respectively) or combined with QS-21 (GLA-LSQ2 or GLA-LSQ3). We recently published prophylactic protection against *M.tb* H37Rv and *M.tb* HN878 using ID93 and lipid-based adjuvants in the mouse model (Baldwin et al., 2021) and hypothesized that these QS-21 containing GLA-liposomal adjuvants could also work therapeutically. An adjuvant containing a monophosphoryl lipid A (MPL) and a neutral liposome containing QS-21 (AS01_E_, GSK Biologicals) has been tested with another subunit TB vaccine antigen, M72, invented by us, and is the furthest along in the TB vaccine development pipeline. M72/AS01_E_ was ~50% effective for a period up to 3 years after the boost immunization, for prevention-of-disease (POD) in a Phase 2b clinical trial (Van Der Meeren et al., 2018; Tait et al., 2019). A vaccine that can boost immune responses generated to either BCG or latent *M.tb* infection will be an important path forward to consider for the development of a vaccine that will prevent disease and transmission. The ID93 + GLA-SE has been shown to boost BCG efficacy in preclinical studies (Bertholet et al., 2010; Kwon et al., 2019).

Our prior studies showed that a robust Th1 immune response, induced by ID93 + GLA-SE, leads to fewer pulmonary lesions and decreased immunopathology when given either prophylactically (Bertholet et al., 2010; Baldwin et al., 2016) or therapeutically (Coler et al., 2013; Larsen et al., 2018). Therefore, we hypothesized that other GLA-containing adjuvants, including an ASO1-like liposomal adjuvant, may also lead to similar outcomes. Immunogenicity was evaluated 4 weeks after the last therapeutic vaccination, and the greatest magnitude of CD4+ Th1 response was observed in mice given the ID93 + GLA-LSQ3 vaccine. Both ID93 + GLA-LSQ vaccines given therapeutically induced a significant polyfunctional CD4 + CD44 + CD154+ T cell response (TNFα, IL-2, and/or IFNγ) compared to unimmunized responses, or other ID93+ GLA liposomal vaccines; however, the ID93 + GLA-LSQ3 included a triple Th1 polyfunctional cytokine response including IFNγ, TNFα, and IL-2 (Figure 1C). Both of the ID93 + GLA-LSQ-containing vaccines led to greater numbers of resident memory T cells within the lung, in addition to increased levels of T cell differentiation shown by increased CD4 T cells expressing PD1 and/or KLRG1. Henao-Tamayo et al. have shown that these markers on T lymphocytes including PD1, in addition to KLRG1, decline over time in *M.tb*-infected mice treated with anti-mycobacterial drugs, indicating that there is a correlation with declining bacterial numbers (Henao-Tamayo et al., 2011). As a result, PD1 and KLRG1 markers were suggested as potential biomarkers for successful chemotherapy against *M.tb* (Henao-Tamayo et al., 2011). These markers have also been studied in *M.tb* infected patients covering different stages of TB, including latent TB infection (LTBI), active TB, and cured TB compared to healthy donors, all with or without prior BCG vaccination (Boer et al., 2016). In cured TB patients, KLRG1+, PD-1+, and CTLA4+ T cells were significantly increased following stimulation with BCG and also to a lesser extent on T cells from actively infected TB patients (Boer et al., 2016). This is in alignment with what we have observed, where PD1+ and KLRG1+ CD4+ T cell responses were increased in the group given the ID93 + GLA-LSQ3 vaccine and drug treatment, which also resulted in statistically significant percent survival compared to the drug treatment only. Interestingly, although the magnitude of the response was greater in the ID93 + GLA-LSQ3-vaccinated animals, the ID93 + GLA-LSQ2 vaccine also led to significant increases in PD1 + KLRG1+ CD4+ T cell responses; however, this group was not found to increase survival over drug treatment. Others have tried strategies to target PD-1, for example, through the use of anti-PD-1 monoclonal antibodies in order to enhance T cell function in *M.tb*-infected NHPs, but this has led to worsened infection and disease (Kauffman et al., 2021). Similarly, *M.tb* infection in PD-1 deficient mice leads to increased proinflammatory cytokine responses in the lung, increases bacterial load, and negatively impacts their survival (Lazar-Molnar et al., 2010). Therefore, the use of host-directed therapy based on a therapeutic vaccine may be a more desirable way to promote balanced immune responses in the context of drug treatment.

We hypothesized that the GLA-liposomal formulations combined with QS-21 would lead to reduced bacterial burden and decreased pulmonary pathology. Our studies show that at the timepoint where lung pathology (measured as the average percentage of lesions within the lung) was observed (4 weeks after the last immunotherapeutic vaccination), there were no significant differences in either bacterial burden or pathology observed between the drug treatment alone or with immunotherapy. The lack of difference between the drug-treatment group and the other groups given immunotherapy showed that the vaccines themselves did not exacerbate the pathology in the lungs early after vaccination. Interestingly, we measured bacterial burden at the time of morbidity (in the survival cohort) and the only vaccines that showed a decrease in lung CFU compared to the unimmunized (drug treatment only) group were the ID93 + GLA-AF and the ID93 + GLA-LSQ2 vaccines (neither of which were shown to be protective by survival measures). In the spleen, significant differences were also observed between the ID93-GLA-LS2 (protected by survival endpoint) and ID93 + GLA-LSQ2 (not protected by survival endpoint) groups, with decreased bacterial load observed in the ID93 + GLA-LSQ2-vaccinated animals.

The main endpoint for therapeutic efficacy in this study was survival. Therapeutic administration of two of the ID93 vaccines combined with liposomal adjuvants was able to enhance survival, including ID93 + GLA-LS2 and ID93 + GLA-LSQ3. Interestingly, QS-21 was needed in combination with ID93 and GLA-LS3 (ID93 + GLA-LS3 did not increase survival over that seen with drug treatment alone). On the other hand, ID93 + GLA-LS2 enhanced survival, whereas the addition of QS-21 to that formulation led to no difference in survival compared to drug treatment alone. The ID93 + GLA-LSQ3 vaccine, given therapeutically, induced significant Th1 responses compared to drug treatment alone, in addition to GM-CSF. A robust polyfunctional CD4 + CD44 + CD154+ Th1 response (IFN-γ, TNFα, and IL-2) compared to drug treatment alone was only: induced by the ID93 + GLA-SE vaccine, however both the ID93 + GLA-LSQ2 and ID93 + GLA-LSQ3 vaccines led to significant single-cytokine producing CD4+ Th1 cells including IFNγ, TNFα, and IL-2, in addition to GM-CSF. Whereas the ID93 + GLA-LS2 vaccine did not elicit a vaccine-mediated CD4+ T cell response, it did induce an *M.tb*-lysate-specific response (albeit not significant), similar to the GLA-LSQ3-and GLA-LSQ2-containing vaccines, that included the production of IFNγ. The ID93+GLA-LS2 vaccine was also the only vaccine to induce a significant percentage of CD4+ regulatory T cells in the lungs 4 weeks after the last immunotherapeutic vaccine was given. This suggests that the ID93+GLA-LS2 vaccine could have a different mechanism of protection compared to ID93+GLA-LSQ3. Moreover, these differences could indicate that GLA (without QS-21) is most effectively formulated in the anionic liposome formulation, whereas QS-21 is optimally formulated in a neutral DOPC-based formulation.

The addition of QS-21 to the GLA-liposomal adjuvants (either neutral or anionic liposomes) also led to increases in the number of non-specific (i.e., non-tetramer stained) resident memory T cells within the lungs and generation of differentiated CD4+ T cells based on CD4+ T cell surface staining with PD1 and KLRG1. Based on the numbers of CD4 + CD44+ T cells, mice immunized therapeutically with ID93 vaccines containing liposomal GLA-LSQ adjuvants induced the highest magnitude of differentiated T cells in the lung, including short-lived effector cells expressing PD1 + KLRG1+ and terminally differentiated cells expressing PD1-KLRG1+. There is new excitement in the numerous ways that vaccine-or *M.tb*.-induced antibody responses may be beneficial for host-induced protective responses. Several functional attributes of antibodies could potentially play a role in protection against *M.tb* which have been described in depth in many excellent reviews, which include antibody-dependent cellular cytotoxicity (ADCC) and antibody-dependent cellular phagocytosis (ADCP) among several other well-characterized functions (Achkar et al., 2015; Lu et al., 2016, 2018; Larsen et al., 2022). In the case of intravenous administration of BCG in rhesus macaques, where six out of 10 of the NHPs were completely protected (with no signs of infection) against *M.tb* (Darrah et al., 2020), an enhanced anti-lipoarabinomannan (LAM) IgM response was induced and measured in the plasma, which negatively correlated with the burden of *M.tb* in the lungs (Irvine et al., 2021). Furthermore, within the bronchoalveolar lavage fluid (BAL), anti-LAM IgM, IgA, and IgG1 antibodies, and other *M.tb*-specific antibodies, were also found to be negatively correlated with *M.tb* burden (Irvine et al., 2021). In a Phase 1 dose-escalation clinical trial in healthy, non-BCG vaccinated individuals, the ID93 + GLA-SE vaccine resulted in higher titers of ID93-specific IgG1 and IgG3 subclasses (Coler et al., 2018). Functional antibody responses were also induced in ID93 + GLA-SE-immunized individuals, including increases in ID93-specific ADCC and ADCP (Coler et al., 2018). In the Phase 2b trial with M72 + AS01_E_, which was approximately 50% effective at prevention of TB disease, anti-M72 titers were measured and detectable through 36 months (Tait et al., 2019). In our current study, regardless of the adjuvant formulation, there were no significant differences in the levels of ID93-specific total IgG, IgG1, and IgG2c in any of the therapeutic vaccines. There was also an antibody response to all of the components that make up the ID93 fusion protein compared to the unimmunized control group. It is important to note that these responses were measured only to the vaccine antigens within the sera of the mice and functional antibody responses were not evaluated. A recent paper by Nziza et al. recently described antibody fingerprints across not only standard antigens, such as lipoarabinomannan (LAM), and purified protein derivative (PPD), but also across 207 other *M.tb* antigens upregulated during hypoxia within the lung, that could potentially distinguish between active and latent TB and could provide use diagnostically (Nziza et al., 2022). This knowledge will be useful to implement in future therapeutic studies in both humans and preclinical models.

These studies show that although we observed no differences in pathology or decreases in *M.tb* bacterial burden, the ID93 therapeutic vaccines, including both ID93 + GLA-LS2 and ID93 + GLA-LSQ3, significantly enhanced survival compared to drug treatment alone while inducing very different immune responses. We have previously shown that the well-established GLA-SE formulation combined with ID93 is effective in both mice and non-human primate models when used in combination with a drug treatment regimen (Coler et al., 2013). Based on the results of this study and on the positive prevention of disease clinical trial results from the *M.tb* vaccine candidate M72/AS01_E,_ which was first developed by our group, we are moving forward with the GLA-LSQ3 adjuvant, which is similar to the AS01_E_ liposome-based adjuvant containing monophosphoryl lipid A (MPL) and QS-21 (GSK). We are currently testing ID93 + GLA-SE (where GLA is mixed in an emulsion rather than a liposomal formulation) in head-to-head experiments with the down-selected ID93 + GLA-LSQ3 vaccine candidate, prioritized in our current work, in two different animal models (mice and guinea pigs) to determine the optimal adjuvant formulation needed to help inform clinical studies on the optimal ID93-based vaccine for use as an adjunct to drug treatment. In a Phase 2a clinical trial, the ID93 + GLA-SE vaccine has recently proven to be safe and immunogenic in adults that have completed treatment against TB (Day et al., 2020).

## Data availability statement

The original contributions presented in the study are included in the article/Supplementary material, further inquiries can be directed to the corresponding author.

## Ethics statement

The animal study was reviewed and approved by the Infectious Disease Research Institute (IDRI) IACUC in accordance with the regulations and guidelines of the IACUC (Protocol Numbers: 2011–5, 2014–9, and 2017–9).

## Author contributions

RC and SB: conceptualization and funding acquisition. RC, SB, SL, VR, BP, BB, BG, and CF: methodology. CF: validation of adjuvants. VR, TP, SL, BP, and BB: formal analysis. RC, SB, VR, SL, TP, BP, BB, BG, CF, and SR: investigation. CF and BP: resources. SB and VR: writing—original draft preparation. RC, SB, VR, SL, TP, BB, BP, CF, and SR: writing—reviewing and editing. All authors contributed to the article and approved the submitted version.

## Funding

This research was funded by the National Institute of Allergy and Infectious Diseases to SB and RC (R01AI125160), IMPAc-TB (75N93021C00029) to RC, and through the University of Washington, Diseases of Public Health Importance T32 Postdoctoral Fellow training grant (AI07509) to SL.

## Conflict of interest

SR is a paid employee of HDT Bio Corp. CF is an inventor on patent applications involving QS-21 purification, GLA-LSQ, and GLA-SE (US 2017/032756; US 2018/049832), CF, SR, and SB are inventors on improved adjuvant formulations comprising TLR4 agonists and methods (EP2811981A1), SR and RC are inventors on patent applications involving ID93 (US 2017/9822152 and 2013/8486414), and SR is on patents involving synthetic glucopyranosyl lipid adjuvants (US 2017/9814772). Shared material may require an MTA or license from the AAHI. BG is was employed by Sana Biotechnology.

The remaining authors declare that the research was conducted in the absence of any commercial or financial relationships that could be construed as a potential conflict of interest.

## Publisher’s note

All claims expressed in this article are solely those of the authors and do not necessarily represent those of their affiliated organizations, or those of the publisher, the editors and the reviewers. Any product that may be evaluated in this article, or claim that may be made by its manufacturer, is not guaranteed or endorsed by the publisher.

## Footnotes

^1^ cdc.gov/tb/topic/treatment/tbdisease.htm
