# Supplementary material for: Therapeutic efficacy against Mycobacterium tuberculosis using ID93 and liposomal adjuvant formulations
Source: Front Microbiol. 2022 Aug 26;13:935444. doi: 10.3389/fmicb.2022.935444 (PMC9459154; doi:10.3389/fmicb.2022.935444)
Supplement: Supplementary file 1 [file Data_Sheet_1.PDF]

Supplementary Material

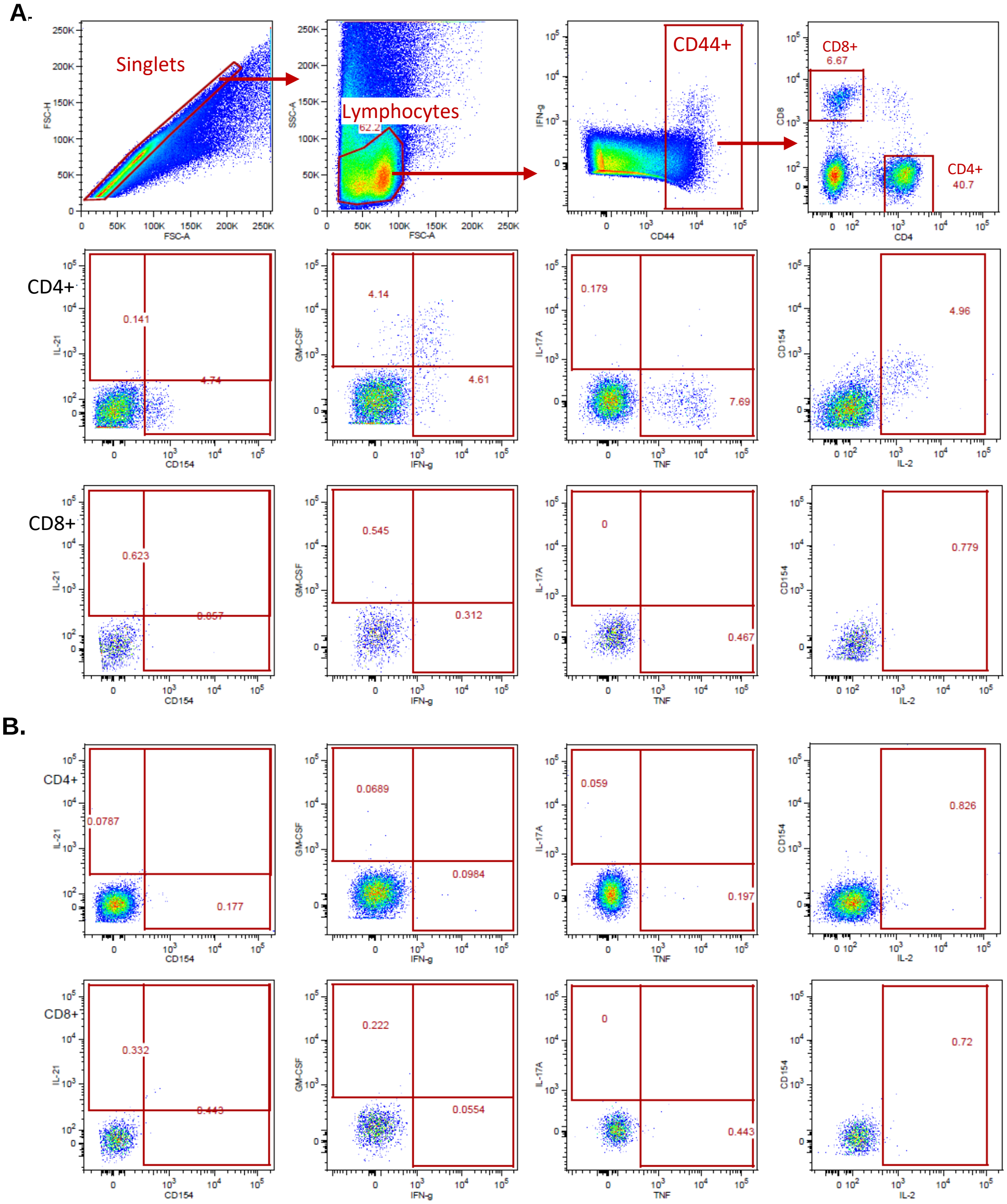

Supplementary Figure 1. Intracellular cytokines gating scheme for (A) CD44+, CD4+ and CD8+ T cells expressing Th1 (IFNγ, TNFα, IL-2) and Th17 cytokines (IL-17A), in addition to IL-21 and GM-CSF (ID93 stimulation shown); (B) medium only culture from same animal shown for comparison.

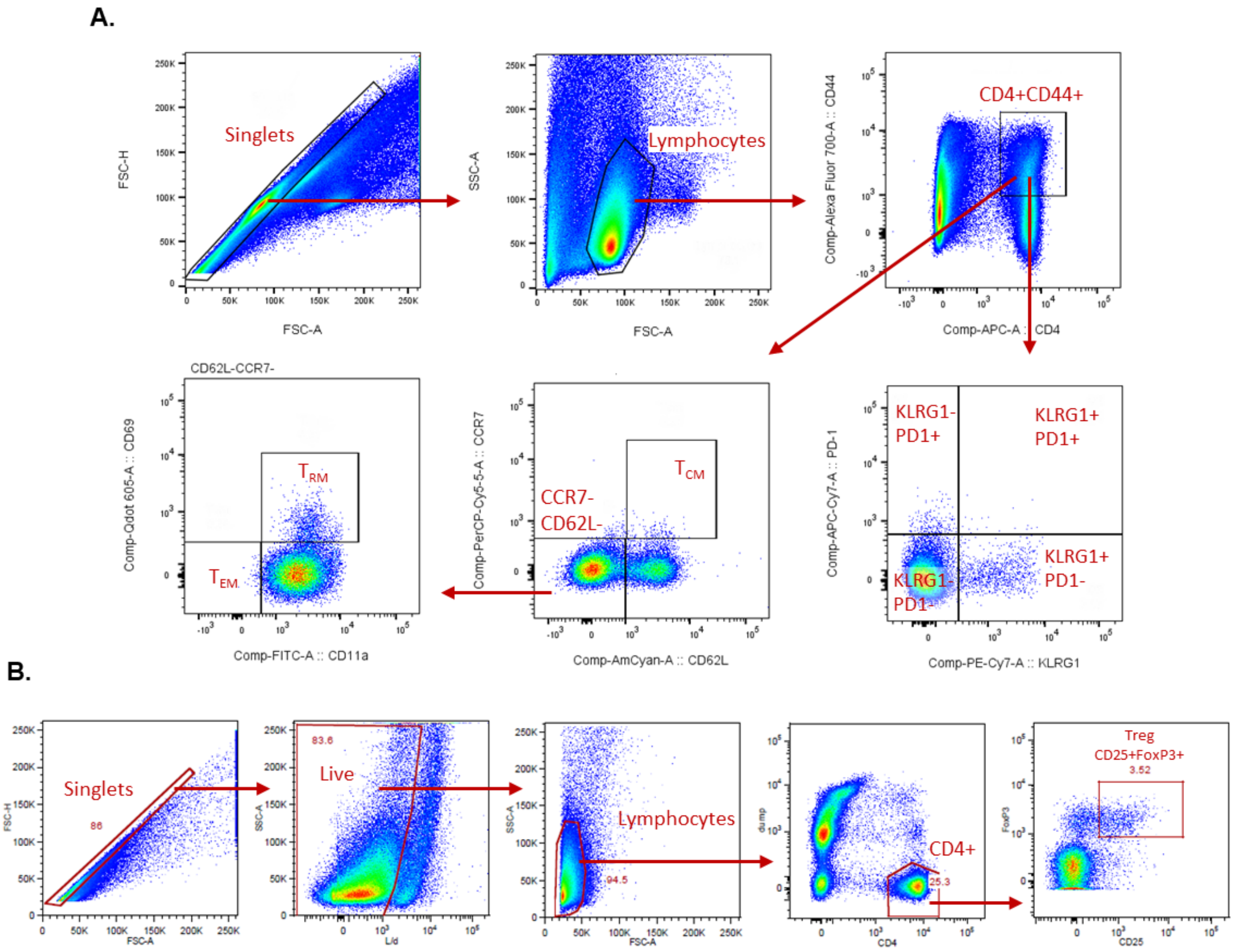

Supplementary Figure 2. Gating schemes for (A) effector memory T cells (Tem), central memory T cells (Tcm), and resident memory T cells (Trm); and (B) CD25+FoxP3+ regulatory CD4+ T cells.

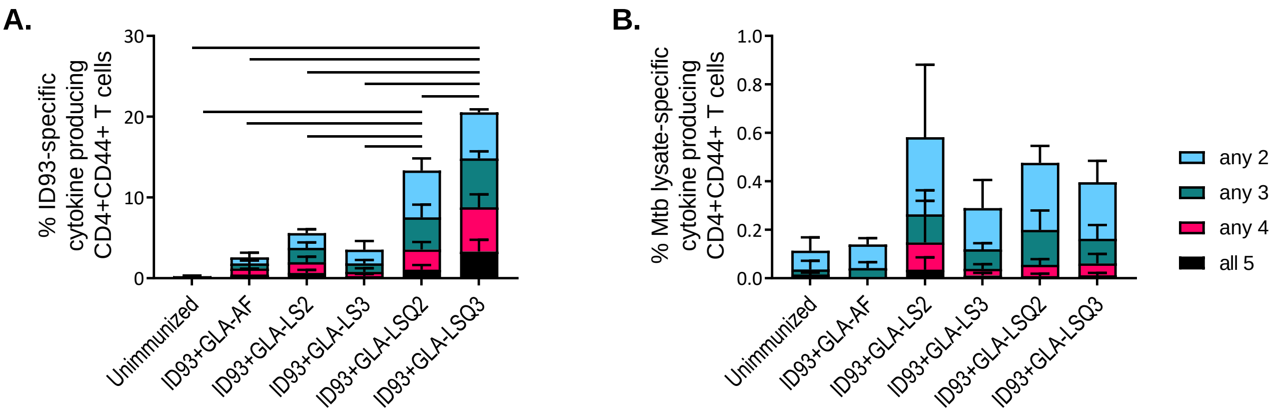

Supplementary Figure 3. Polyfunctional CD4+CD44+ T cells in female SWR/J mice after treatment with drug and therapeutic ID93 vaccines. (A) Percent frequency of CD4+CD44+ ID93-specific stacked cells producing 2 or more cytokines; the bars indicate statistical significance, where

p<0.05 using one-way ANOVA with Bonferroni's multiple comparison correction; (B)) Mtb lysate-specific CD4+CD44+CD154+ polyfunctional cytokine producing cells, no significance between groups. Boolean gating was done including CD154, GM-CSF, IFNγ, IL-2, and TNFα, then summed by number of cytokines produced.

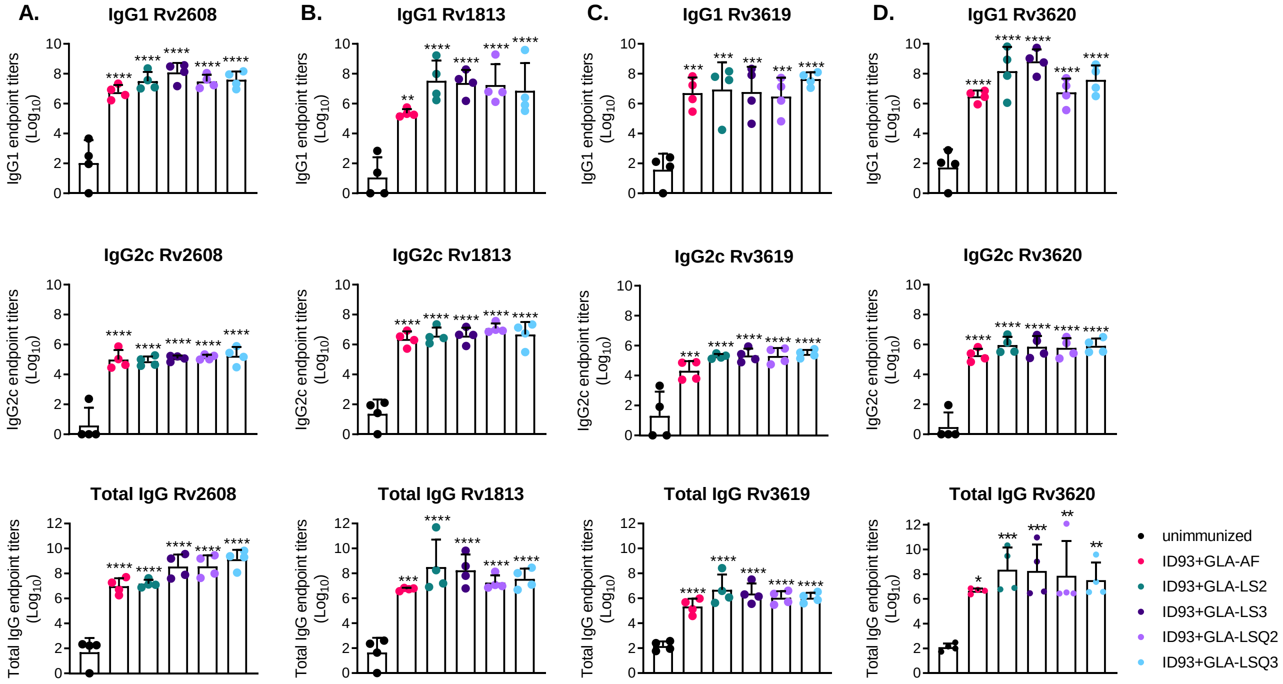

Supplemental Figure 4. ID93 component-specific IgG subclass responses (IgG1, IgG2c, and total IgG) to in SWR mice following therapeutic vaccination with different formulations of GLA. Columns represent endpoint antibody responses to each component of ID93: (A) Rv2608; (B) Rv1813; (C) Rv3619; and (D) Rv3620. All of the adjuvanted therapeutic vaccinations induced significant levels of each subclass of IgG. Dots represent individual mice, bars represent the Mean.

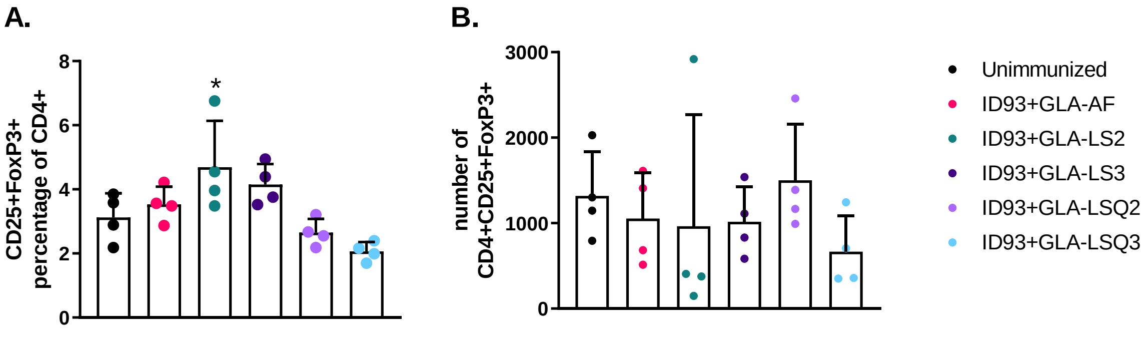

Supplemental Figure 5. Regulatory T cells in the lung. (A) percentage of CD4+CD25+FoxP3+ cells; and (B) number of CD4+CD25+FoxP3+ are represented 18 weeks after infection with *Mycobacterium tuberculosis* H37Rv (4 weeks after the last immunizations with the ID93 therapeutic vaccines). Dots represent individual mice, bars are Mean, and whiskers SD.

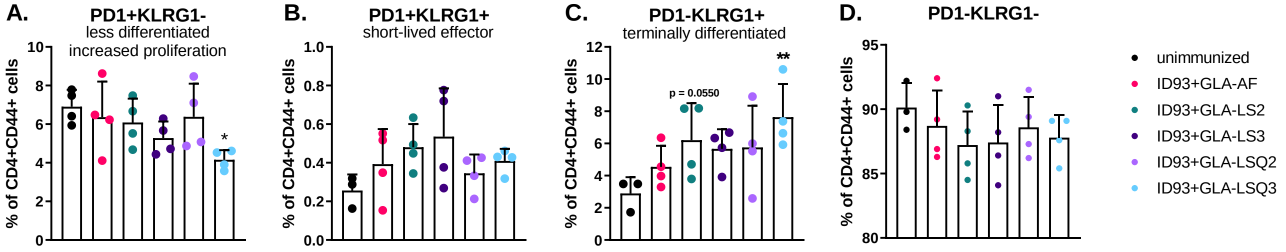

Supplemental Figure 6. Multiple stages of differentiated CD4+ T cells in the lungs in mice immunized with ID93 combined with GLA liposomes (either neutral or anionic) combined with QS-21. SWR mice were challenged with Mtb H37Rv, then treated for 8 weeks with Isoniazid, Rifampin, and Pyrazinamide in the drinking water, followed by treatment with ID93 therapeutic vaccines 4 weeks after the start of drug treatment 3 times, 3 weeks apart. Four weeks after the last therapeutic vaccination, mice (n=4 mice/group) were euthanized, and lungs were harvested for flow cytometric analysis. Dots represent individual mice, bars are Mean, and whiskers SD. Asterisks indicate statistical significance (*P*<0.05) in mice given therapeutic immunization versus no immunization, using one-way ANOVA with Bonferroni's multiple comparisons correction. The percent frequency of CD4+CD44+ cells expressing PD1 and KLRG1 markers are as shown: (A) PD1+KLRG1- (less differentiated, increased proliferation); (B) PD1+KLRG1+ (short-lived, cytokine-producing, effector T cells); (C) PD1-KLRG1+ (terminally differentiated, cytokine-producing T cells); (D) PD1-KLRG1-.

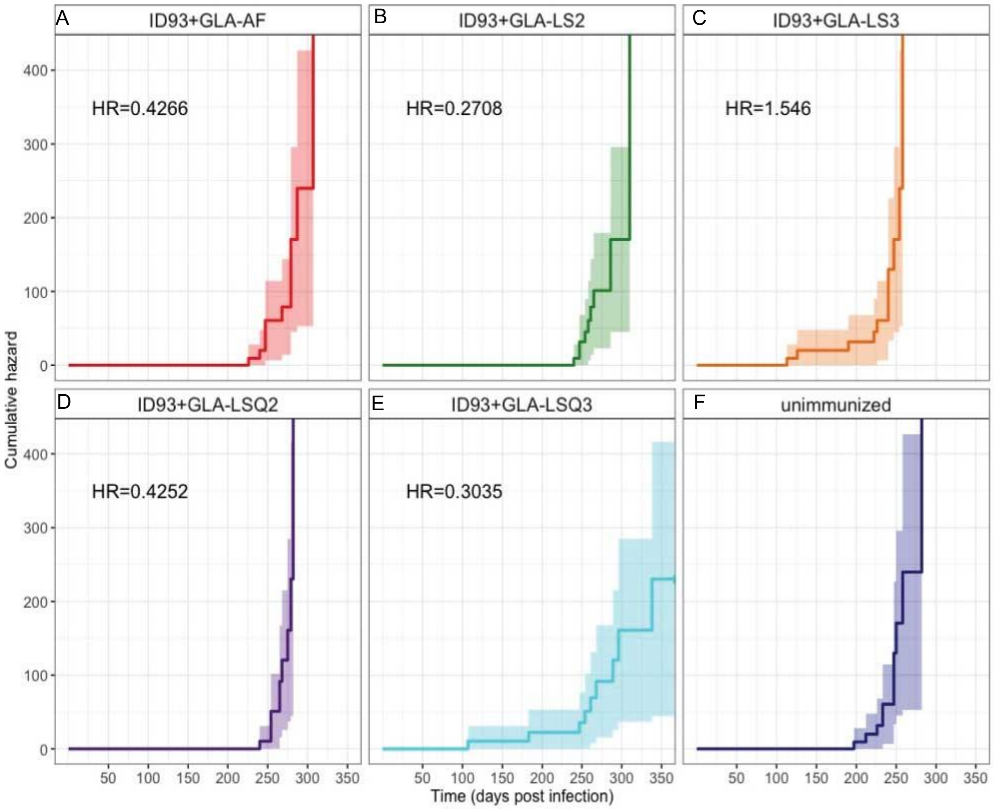

Supplemental Figure 7. Kaplan-Meier cumulative hazard curves for each intervention. SWR mice were challenged with Mtb H37Rv, then treated for 8 weeks with Isoniazid, Rifampin, and Pyrazinamide in the drinking water, followed by therapeutic immunization with ID93 vaccines 4 weeks after the start of drug treatment 3 times, 3 weeks apart. 95% CI is indicated in the shaded region on each side of the curve. Hazard ratios (HR) were determined using Cox Proportional Hazard models and are relative to the unimmunized group. N=11 mice per group, except for ID93+GLA-LSQ2 and ID93+GLA-LSQ3 (n=9 mice/group) (A) Drug treatment + ID93+GLA-AF; (B) Drutreatment + ID93+GLA-LS2; (C) Drug treatment + ID93 + GLA-LS3; (D) Drug treatment + ID93 + GLA-LSQ; (E) Drug treatment + ID93 + GLA-LSQ3; (F) Drug treatment only (unimmunized).

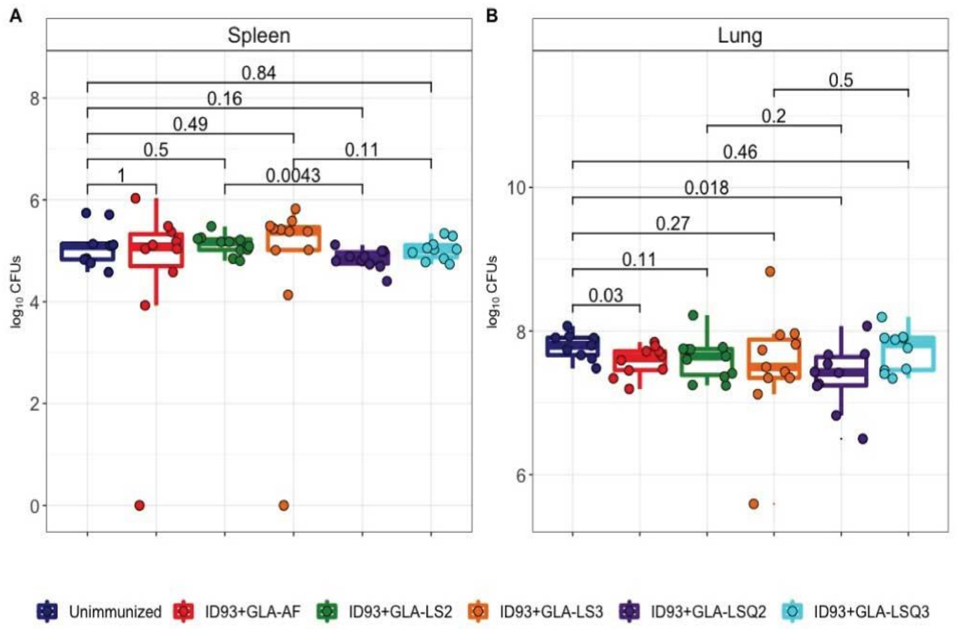

Supplemental Figure 8. Supplemental Figure 8. Individual colony forming units (CFU) at death for each intervention group. (A) Bacterial CFU in the spleen; (B) Bacterial CFU in the lung. Indicated *P* values were calculated using a Wilcoxon Rank Sum test. Boxplots display the median value and the box spans the inter-quartile range(IQR; the 25th and 75th percentiles), with the whiskers extending to 1.5 * IQR.
